# A miniaturized sandwich immunoassay platform for the detection of protein-protein interactions

**DOI:** 10.1186/1472-6750-10-78

**Published:** 2010-10-28

**Authors:** Qiongming Liu, Qing Chen, Jian Wang, Ying Zhang, Ying Zhou, Cong Lin, Wei He, Fuchu He, Danke Xu

**Affiliations:** 1 State Key Laboratory of Proteomics, Beijing Proteome Research Center, Beijing Institute of Radiation Medicine, Beijing 102206, China; 2 Institutes of Biomedical Sciences, Fudan University, Shanghai, 200032, China; 3 Key Lab of Analytical Chemistry for Life Science (MOE), School of Chemistry and Chemical Engineering, Nanjing University, Nanjing, 210093, China

## Abstract

**Background:**

Analysis of protein-protein interactions (PPIs) is a valuable approach for the characterization of huge networks of protein complexes or proteins of unknown function. Co-immunoprecipitation (coIP) using affinity resins coupled to protein A/G is the most widely used method for PPI detection. However, this traditional large scale resin-based coIP is too laborious and time consuming. To overcome this problem, we developed a miniaturized sandwich immunoassay platform (MSIP) by combining antibody array technology and coIP methods.

**Results:**

Based on anti-FLAG antibody spotted aldehyde slides, MSIP enables simple, rapid and large scale detection of PPIs by fluorescent labeling anti-myc antibody. By analyzing well-known interacting and non-interacting protein pairs, MSIP was demonstrated to be highly accurate and reproducible. Compared to traditional resin-based coIP, MSIP results in higher sensitivity and enhanced throughput, with the additional benefit of digital read-outs. In addition, MSIP was shown to be a highly useful validation platform to confirm PPI candidates that have been identified from yeast two hybrid systems.

**Conclusions:**

In conclusion, MSIP is proved to be a simple, cost-saving and highly efficient technique for the comprehensive study of PPIs.

## Background

Protein-protein interactions (PPIs) are ubiquitous to virtually every cellular process. There have been a lot of interest in systematically mapping PPI networks for better understanding of the mechanisms of biological processes. Various approaches, including solution biochemistry using purified proteins, immunoprecipitations (IP), tandem affinity purifications (TAP), yeast two-hybrid (YTH) and phage display have been developed for characterization of PPIs. Characterization of huge networks of proteins requires methods that are amenable to high-throughput (HT). At present, the yeast two hybrid (YTH) method, affinity purification followed by mass spectroscopy (AP/MS) and Mammalian Two Hybrid (MTH) assay have been successfully employed to map PPIs at the proteome scale [1-9]. However, these HT methods always lead to high rates of false positive/negatives [10] and the coverage is low, which complicates the interpretation of the data. This complication is highlighted by the fact that comparable efforts from multiple laboratories using either the YTH system or AP/MS have obtained only a small overlap in the number of positive interactions identified, regardless of the method used and despite testing similar gene sets [11,12]. This lack of concordance suggests that a more accurate HT method for PPI detection is required.

Co-immunoprecipitation (coIP) is one of the most reliable techniques to study PPIs *in vivo *and is the most widely used method to confirm interactions identified by YTH or AP/MS methods [13-15]. To perform coIP, an antibody against a specific protein target is coupled to a resin via protein A or G. Incubation of the antibody complex with a mix of proteins, such as a cellular lysate, results in specific binding to the target protein which then can be immunoprecipitated from solution by centrifugation. Protein components in these precipitated complexes are then denatured and visualized by Western blotting. This affinity-based molecular pull-down method, facilitated by epitope tagging of recombinant proteins, has enabled detailed investigation of protein expression, function and interaction patterns. Traditional resin-based coIP using affinity resins, however, relies on multiple binding, washing and elution steps that are performed in individual microfuge tubes, and require repeated centrifugation, aspiration, and suspension steps. These laborious and time-consuming steps erode the overall analysis throughput. A simple, streamlined procedure for handling large numbers of PPI candidates, enable us to validate interactions detected by other methods including YTH and AP/MS or can be used as a stand alone system to detect PPIs is therefore highly desirable.

Biochips are solid surfaces arrayed with test-sites in high spatial density that were developed as miniaturized, robust platforms for the high-throughput study of biomolecules. We report here a miniaturized sandwich immunoassay platform (MSIP) for the detection of PPIs that combines antibody microarray technology with a traditional coIP. Nanoliter volumes of anti-flag antibody are printed onto aldehyde slides in an array format, providing a platform for the analysis of immunoprecipitates from small amounts of crude cell lysate containing FLAG-bait and myc-prey. Detection is achieved via fluorescence imaging, using tagged anti-myc antibody and an array scanner. Compared to traditional resin-based coIP, MSIP requires much smaller amount of cell lysates, allows for a large number of samples to be studied en masse without the need for further manipulation of the slides, and eliminates the need for further immunofluorescence staining or enzymatic amplification.

## Results

### Overview of MSIP

**Figure 1:**
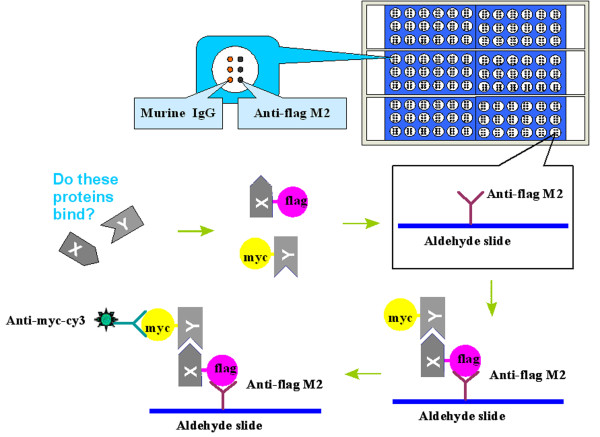
**Overview of the bait-prey approach of MSIP**. A 'bait' protein is tagged with FLAG while a 'prey' is tagged with myc. Following transfection and expression of the FLAG- and myc-tagged constructs, cells are lysed and the crude cell lysate is incubated on an anti-FLAG printed aldehyde slide. The FLAG-tagged bait (FLAG-X) is captured by the antibody on the surface of the aldehyde slide, co-capturing any interacting proteins. Myc-tagged prey (myc-Y) interacting with bait is detected by cy3-taged monoclonal anti-myc antibody.

MSIP detection of mammalian PPIs was developed to identify protein binding partners in high-throughput (Figure 1). This bait-prey approach utilized FLAG- and c-myc-tagged interacting proteins. Following transfection and expression of the FLAG and c-myc constructs in mammalian cells, cells were lysed and coIP was performed on the slide surface. The FLAG-fusion was captured by anti-FLAG antibody, while interacting proteins co-captured were identified by fluorescent anti-myc-cy3 antibody. To achieve large scale analysis, a multiplexed array containing 9 × 12 sub-arrays was fabricated on six slides assembled in a custom designed holder, allowing for dismantling of the slides from the holder and analysis of the slides individually by a universal fluorescent scanner. This design supports large scale analysis, as analytes may be transferred to the multiplexed array from 96 microwell dishes.

### Evaluation of the specificity and reproducibility of MSIP

**Figure 2:**
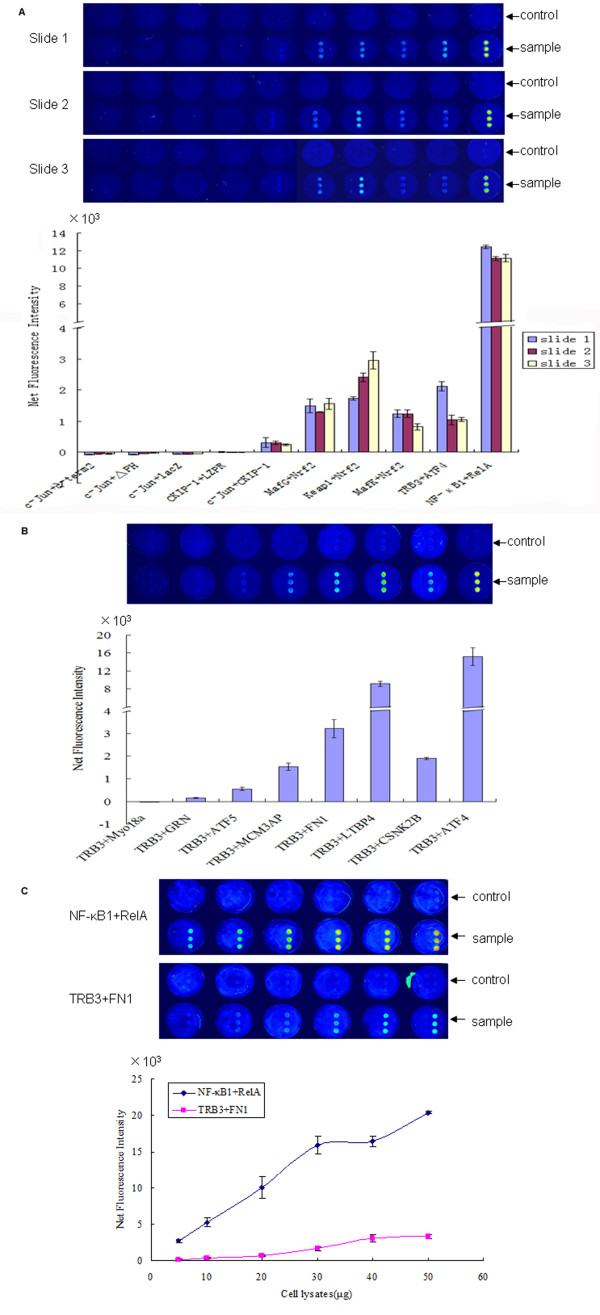
**Evaluation of the capability and reproducibility of MSIP**. HEK293 cells were cotransfected with FLAG-bait and myc-prey constructs. For negative controls, each myc-prey was cotransfected with pflag-CMV-2. Cell lysates were incubated in the frame of the anti-FLAG spotted slides at room temperature for 1 h. FLAG-bait and any interacting proteins were captured on the surface of the slides. Myc-prey was detected by using monoclonal anti-myc-cy3 (1:200). The image was scanned by a fluorescence scanner, and the NFI of each PPI was calculated. A) Six pairs of well-characterized interacting proteins and 4 pairs of known non-interacting proteins were analyzed by MSIP. The analysis was performed on each of three slides assayed on different days with equal amounts of cell lysates derived from independent transfections. B) eight pairs of protein partners using the same bait were analyzed by MSIP with a slide. Equal amount of cell lysates were incubated on the anti-flag spotted slides. C) Different quantities of the same cell lysates were used to analyze the NFI of two pairs of previously identified positive PPIs by MSIP with two slides.

To demonstrate the specificity of MSIP detection of PPIs, 6 pairs of well-characterized interacting proteins and 4 pairs of known non-interacting proteins were tested on the surface of anti-flag spotted slides (Additional file 1: Table S1) [16-20]. Nonspecific adsorption of bait and prey were reduced by spotting murine IgG in the same frame as the capture antibody. The constructs of each interaction pair were expressed in HEK293 cells, and lysates were incubated in a frame on the surface of anti-FLAG spotted slides. Cell lysates containing myc-prey but not FLAG-bait were used as negative controls. Fluorescent intensity values were determined by subtracting the values from the murine IgG spots. The net fluorescence intensity of each PPI was calculated as the fluorescence intensity of each bait (FLAG-fusions) and prey (myc-fusions) subtracted by the corresponding negative control (myc-tagged prey in the absence of FLAG-tagged bait). This proof of principle MSIP experiment indicates that the NFI values of the well-characterized 6 pairs of binding partners ranged from 243.00 to 12412.33, while those of the negative controls ranged from -84.67 to 6.33 (Additional file 1: Table S1). Thus, MSIP correctly identified each of the 6 pairs of binding partners as positive and each of the 4 non-interacting proteins as negative (Figure 2A).

In a second confirmation of the specificity of MSIP, 8 pairs of interaction candidates that share the same bait were analyzed (Additional file 1: Table S2). These interaction partners were identified by YTH in our lab and seven of these have been confirmed by resin-based coIP [21]. Each protein-protein interaction was assessed by MSIP following incubation with equivalent amounts of total cellular lysate. While no detectable interaction between TRB3 and Myo18a was observed, MSIP detected positive interactions between each of the remaining protein pairs of various fluorescence intensities (Figure 2B). These results are consistent with our previous work, which identified PPI amongst each of the seven positives by coIP while did not detect interaction of TRB3 with Myo18a [21]. The NFI values for the seven positive interactions varied from 166.67 to 15171.17 (Additional file 1: Table S2).

To test the reproducibility of MSIP, the analysis was performed on each of three slides assayed on different days with equal amounts of cell lysates derived from independent transfections (biological replicates). Both the positive and the negative signals were reproduced accordingly. As most of the FI values for the negative interaction pairs are negative number, it was meaningless to calculate their coefficient of variation (CV). The CV for the 6 positive interaction pairs were ranged from 6% to 43% (13% for c-Jun/CKIP-1; 9% for MafG/Nrf2; 26% for Keap1/Nrf2; 22% for MafK/Nrf2; 43% for TRB3/ATF4; 6% for NF-κB1/RelA) (Additional file 1: Table S3). To test the technical replicacy of the assay, different quantities of the same cell lysates were analyzed using two pairs of interactions (NF-κB1/RelA and TRB3/FN1). Image and NFI values (Additional file 1: Table S4) increased in a dose-dependent manner with increasing quantity of cell lysates (Figure 2C). These results demonstrate that the MSIP results in both accuracy and reproducibility.

### Evaluation of the NFI of each PPI and determination of its cutoff value

**Figure 3:**
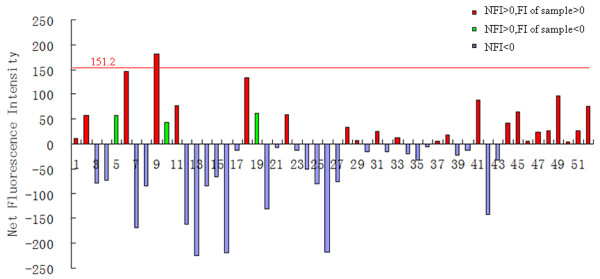
**Determination of the cutoff NFI value for detection of PPI**. DNA constructs of 52 pairs of flag-bait and myc-prey were randomly coupled and transfected into HEK293 cells. For negative controls, each myc-prey was cotransfected with pflag-CMV-2. Cell lysates were incubated on 6 anti-FLAG spotted slides, and PPI were detected using monoclonal anti-myc-cy3 (1:200). Following scanning, no obvious interaction signals were detectable, thus each were considered as non-interacting protein partners. Negative FI and NFI values were discarded, and the cutoff NFI value of 151.2 was calculated as the mean plus two times the standard error of the positive values (red column).

Determination of a cutoff NFI value to assess positive PPIs would allow for rapid large-scale analysis of samples without the need to manually view fluorescent images. In order to determine a suitable NFI cutoff value, 52 pairs of flag-bait and myc-prey were randomly coupled and their interactions were investigated by MSIP. Confocal laser fluorescence scanning of the slides determined that there were no obvious interaction signals. Therefore, these protein partners were considered non-interacting. FI values were recorded and NFI values were calculated (Additional file 1: Table S5). To calculate the cutoff NFI value, negative FI and NFI values were eliminated from the data set (Additional file 1: Table S5, light gray and dark gray shaded boxes, respectively). The cutoff value was set as the sum of the mean of the NFI remaining values plus double the standard error (cutoff NFI value = $\overset{-}{\text{x}}$ + 2SD), and was calculated to be 151.2 (Figure 3). Thus, PPI candidates are considered positive only if their FI and NFI value are both positive, and if the NFI value lies above the cutoff value of 151.2. This strict standard is set up to reduce the detection of non-specific binding proteins.

### Comparison of the MSIP with traditional resin-based coIP

**Figure 4:**
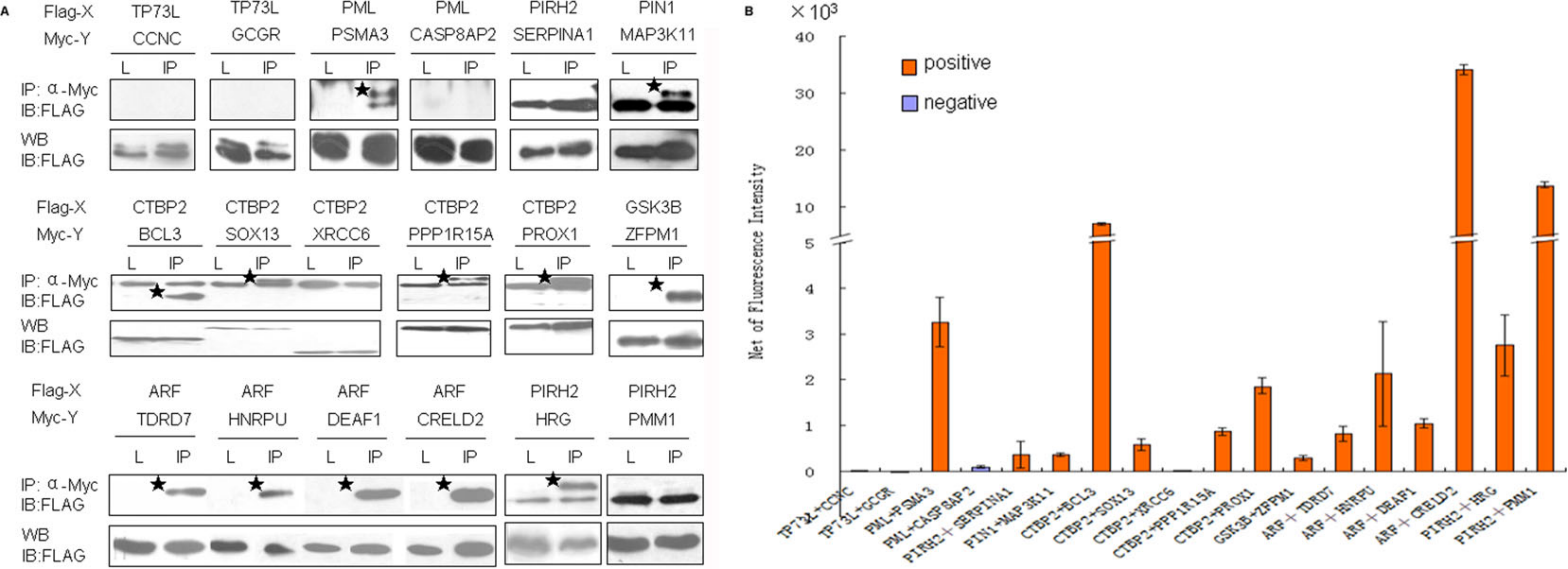
**Comparison of MSIP with resin-based coIP using 18 pairs of interaction candidates identified by YTH**. HEK293 cells were cotransfected with FLAG-bait and myc-prey constructs or negative controls. Protein-protein interactions were analyzed by resin-based coIP and MSIP. (A) The samples were probed for interaction by resin-based coIP via western blotting. Stars indicate protein-protein interactions. (B) Samples were probed for PPI by MSIP with 2 slides using monoclonal anti-myc-cy3 (1:200). The slides were scanned by a fluorescence scanner, and the NFI of each PPI was calculated. Flag-X, flag-tag bait; Myc-Y, myc-tag prey; IP, immunoprecipitation; IB, immunoblotting; WB, western blotting; L, lysate.

Systematic mapping of all PPIs occurring within the liver, which is a primary goal of the Human Liver Proteome Project (HLPP), is being performed by YTH screening in our lab [22]. To systematically evaluate the confidence of the resulting PPI data, traditional resin-based coIP was the most common method for data verification. Due to the laborious and time-consuming procedures for traditional resin-based coIP, we sought to determine if the rapid MSIP procedure could be utilized with similar overall efficacy. To verify the PPI data obtained by YTH screening, 18 pairs of candidate interactions with high confidence (Supplementary table 6) were analyzed by MSIP. DNA constructs of each interaction pair were expressed in HEK293 cells and PPI were determined by both MSIP and resin-based coIP (Figure 4). While twelve of these positive PPIs (66.7%) were confirmed by resin-based coIP, MSIP identified each of those twelve plus an additional two PPIs for a total of fourteen (77.8%). These results demonstrate the efficiency of MSIP is at least comparable to resin-based coIP. While an explanation for the higher positive rate by MSIP is unknown, we speculate that it may be due to either higher sensitivity of MSIP or less potential interaction interference by denatured immunoprecipitating antibody heavy or light chain during Western blot analysis, which occurs during traditional resin-based coIP but not MSIP. In conclusion, MSIP is much simpler, more rapid, large scale, highly sensitive and cost-effective as compared to the traditional resin-based coIP (Additional file 1: Table S7).

### Application of MSIP to verify PPIs from YTH system

**Figure 5:**
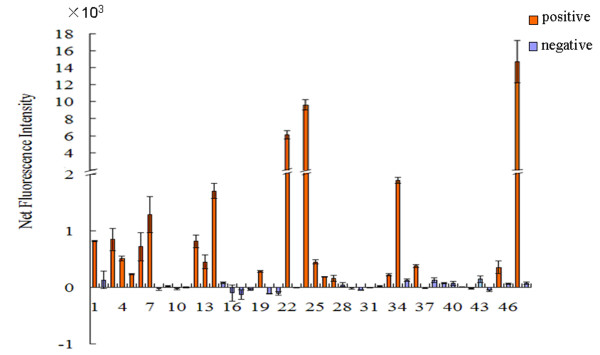
**Application of MSIP for verification of PPI candidates identified by YTH**. Forty eight pairs of interaction candidates, listed in Additional file 1: Table S8 with lane numbers corresponding to their order in the table, were identified by YTH. HEK293 cells plated in 96-well dishes were cotransfected with FLAG-bait and myc-prey constructs or negative controls. Cell lysates were incubated in the frames of the anti-FLAG spotted slides (six slides were used), and protein-protein interactions were detected by monoclonal anti-myc-cy3 antibody (1:200). The slides were scanned by a fluorescence scanner, and the NFI of each PPI was calculated.

We further applied MSIP to verify a pool of candidate interactions identified by the YTH system (Additional file 1: Table S8). 48 pairs of interaction candidates were randomly selected and their expression in HEK293 cells was confirmed by protein microarray using anti-FLAG -Cy3 and anti-Myc-Cy3. The interactions were analyzed by MSIP using a multiplexed array system with 96 analytes consisting of 48 samples and their corresponding control, and the interaction signals were represented by NFI values. We found that 21 pairs of the candidate interactions generated positive FI and NFI values, and the NFI were above the cutoff value, suggesting these PPIs are positive. The remaining 27 pairs, whose NFI values scattering around zero and smaller than the cutoff value, were determined as non-interacting protein pairs (Figure 5). In sum, a total of 66 pairs of candidate interactions identified by YTH screening were analyzed by MSIP. Using the established criteria, 35 pairs of these interactions were considered positive, resulting in a positive ratio of 53.0% among the interactions from YTH system, comparable to the historic positive ratio found in the literature [6,13]. These results demonstrate that MSIP provides a highly useful validation platform for the YTH system.

## Discussion

We have adapted the existing coIP PPI assay, traditionally performed in individual microfuge tubes, to a MSIP for rapid analysis of PPIs at a large scale. As demonstrated here, MSIP displays high efficiency and good reproducibility, and provides digital readouts useful for large-scale analysis. This method has several advantages over the traditional resin-based coIP, including requiring significantly smaller quantities of both antibody and protein samples (cell lysate from 96-well dishes is enough), and greatly reducing experimental cost. Additionally, fewer pipetting steps are involved in this platform, giving fewer sources of operation error and enhancing reproducibility. Moreover, detection of bound protein can be achieved directly on the slides by fluorescence imaging, yielding digital data without the need of detection by Western blotting. The nature of this slide-based assay allows for very rapid washing steps, greatly reducing the time required compared to traditional resin-based coIP, thus the number of samples which can be assayed daily is increased greatly. Spotting mouse IgG on the same frame as that of the capture antibody also permits side-by-side analysis of controls with the same sample. Finally, covalent coupling of the antibody to the surface of the slide allows complete removal of wash buffer without concern of removing samples during the assay. Loss of resin at wash steps using traditional coIP makes resin a less quantitative method compared to the slide-based method. Based on these features, MSIP provides a rapid method for the study of large numbers of samples and numerous variables such as ranges of cell lysate, side-by-side analysis of controls, replicate samples, optimization of reaction conditions, and different time points after treatment in the study of dynamics of PPIs.

Previously, Barrios-Rodiles M and colleagues described a luminescence-based method (LUMIER) for the HT detection of mammalian PPIs [23]. Although the method has been successfully applied to map the dynamic signaling network of transforming growth factor-β, the coIP was performed in microwell using magnetic beads and multiple washing steps were involved. The MSIP, on the other hand, allows very rapid washing steps without the risk of losing samples, as anti-flag antibody is covalently coupled to the aldehyde slide surface. Additionally, Existing FLAG- and myc-tagged bait and prey can be easily incorporated in the MSIP assays. FLAG^® ^96-well Immunoprecipitation system developed by Sigma also provides a validation platform for YTH in an HT format [24]. However, enzyme-based detection via ELISA or Western blotting complicates its handling. In comparison with the Sigma system, the MSIP requires less quantities of antibody and no detection substrates, and nonspecific adsorption can be easily reduced by spotting murine IgG in the same frame as the capture antibody. Further, because fluorescence signals are intensified on the aldehyde slides, the sensitivity of the MSIP is also much higher. Certainly, like most of other techniques, MSIP also has some limitations. Due to possible variables in the reagents (different antibody batches, different levels of transfection efficiencies etc), the cutoff value should be confirmed for each experiment. However, separately determination of the cutoff value with large number of negative bait-pray pairs for each experiment is unpractical. It had better include a set of known positive and negative interaction pairs in each experiment. Such controls would guarantee the proper functioning of the assay. In addition, a pair of known and preferentially weak interaction should be included in each experiment to confirm the threshold. Secondly, for practical reasons, the generation of the transfected cell lysates cannot be performed in a micro-well format larger than the 96-well format. In addition, only 18 bait-prey combinations can be tested per slide, which hamper the applications of MSIP for high throughput assay.

## Conclusion

We have developed a MSIP that exhibits unprecedented efficiency and can be adapted with ease for any PPI analysis. The MSIP combines the advantages of traditional coIP with the efficacy of antibody microarrays, saving considerable time and reagents. The relative ease in analyzing multiple samples in parallel makes MSIP particularly well suited for large scale study or validation of a large number of PPIs.

## Methods

### Reagents, antibodies and plasmids

Complete protease inhibitor was obtained from Roche (Basel, Switzerland). All restriction enzymes and Taq polymerase were obtained from TakaRa (TakaRa, Japan). Monoclonal ANTI-FLAG^® ^M2 antibody, monoclonal ANTI-FLAG^® ^M2-Cy3 antibody, and monoclonal Anti-c-Myc-Cy3 antibody were obtained from Sigma-Aldrich (St Louis, MO). c-Myc monoclonal antibody was obtained from Clontech (Palo Alto, CA). Mouse IgG was obtained from ZhongShan Goldenbridge Biotechnology (China). Cy5-conjugated goat anti-rabbit IgG was labeled in the lab. CSS-100 silylated slides (Aldehyde) were obtained from CEL Associates, Inc. (CEL Associates Inc., Pearland, Texas, USA). Genes for proteins with suspected interaction partners were cloned into pFLAG-CMV-2 and pCMV-myc expression vectors for transient expression in mammalian cells. ORF of different proteins contained the same protein domains as used for the YTH.

### Cell culture and transfection

Human embryonic kidney HEK293 cells were cultured in DMEM (Invitrogen) supplemented with 10% FBS (Hyclone), penicillin, streptomycin and glutamine. One day before transfection, 0.75~3 × 10^4 ^cells in 100 μl DMEM without antibiotics were plated in the 96-microwell format, so that cells will be ~90% confluent at the time of transfection. Total of 200 ng DNA constructs with half of each plasmid were transfected. Transfections were performed with Lipofectamine 2000 (Invitrogen, CA) according to the manufacturer's instructions.

### Preparation of aldehyde slides spotted with antibodies

CSS aldehyde slides (CEL Associates Inc., Pearland, Texas, USA) were spatially separated onto 3 × 6 frames by a removable waterproof stick-film containing 3 × 6 wells. The waterproof stick-film is manually sticked on the surface of the slide before used. Antibody printing onto the slides was performed by a Smart Arrayer-48 spotting robot (CapitalBio, Beijing, China), mounted with an ArrayIt micro spray pin from TeleChem (Sunnyvale, CA, USA). 1.0 mg/ml anti-flag antibody and 1.0 mg/ml normal murine IgG (both diluted with 1.0 mg/ml BSA in TBST) were printed in spots of 0.4 mm in diameter and 1.5 mm intervals (center to center) with an approximately 10 nL spotting volume. During spotting, humidity and temperature in chamber were maintained at 40% and 20°C, respectively. After the printing process, all slides were incubated overnight at 4°C to allow maximum binding of antibody to the aldehyde slide surface.

### Traditional resin-based coimmunoprecipitation

For general cell lysis and co-immunoprecipitation of Flag-X and the candidate interactor Myc-Y, HEK293 cells were transfected with indicated expression vectors by Lipofectamine 2000. After 30 h, cells were harvested and lysed in EBC buffer [50 mM Tris-Cl (pH8.0), 120 mM NaCl, 0.5%(V/V) NP40, 1 mM EDTA] supplemented with 50 μg/ml PMSF and protease inhibitor cocktail (Roche, Basel, Switzerland) at room temperature for 10 min. After centrifugation (4°C, 12,000 rpm, 10 min) the supernatant was used immediately. Immunoprecipitations were performed using normal IgG (for preclear), anti-Myc and protein A/G-agarose (Santa Cruz, CA) at 4°C. The lysates and immunoprecipitates were detected by Western blot using the indicated primary antibodies and appropriate secondary antibody, followed by measurement with SuperSignal chemiluminescence kit (Pierce).

### Miniaturized sandwich immunoassay of PPIs

HEK293 cells plated in 96-well dishes were manually transfected with indicated expression vectors with Lipofectamine 2000. After 30 h, cells were harvested and lysed in 20 μl EBC buffer, as described. The concentration of the cell lysates were determined by Bradford method. To make sure that the recombinant constructs are expressed in HEK293 cells, crude cell lysates were spotted onto the aldehyde slides by noncontact printing, and the expression level of bait and prey were detected on the slides using anti-FLAG-Cy3 and anti-myc-Cy3 respectively. The antibody spotted aldehyde glass slides were blocked by incubation with 10 mg/ml BSA in TBST [20 mM Tris-Cl (pH 8.0), 150 mM NaCl, 0.05% (V/V) Tween 20] at room temperature for 1 h. After blocking, slides were rinsed with TBST for 3 times. 20 μl of cell lysate was transferred into one frame on the slide surface and incubated for 2 h at room temperature. The slides were washed with TBST for 3 times to remove unbound proteins. Protein interacting partners were then detected with monoclonal anti-myc-cy3 (1:200) by incubation in a humid, dark chamber for 45 min. After 3 additional washes with TBST, the frames were removed and the slides were air-dried prior to imaging.

### Signal detection and analysis

To collect the fluorescence signal in the MSIP, slides were scanned using a confocal laser fluorescence scanner Luxscan-10K/A (CapitalBio, Beijing, China) with a resolution of 10 μm per pixel. Laser power and photomultiplier gain were both set to 70%. Image analysis was carried out with Spot Data Pro 2.0 (CapitalBio, Beijing, China). The median pixel values of feature and background were used as the signal and background intensities, respectively. The net fluorescence intensity of each PPI was calculated as the fluorescence intensity of each bait (FLAG-fusions) and prey (myc-fusions) subtracted by the corresponding negative control (myc-tagged prey in the absence of FLAG-tagged bait).

## Abbreviations

PPIS: protein-protein interactions; COIP: coimmunoprecipitation; MSIP: miniaturized sandwich immunoassay platform; IP: immunoprecipitations; TAP: tandem affinity purifications; YTH: yeast two hybrid; MS: mass spectroscopy; HT: high-throughput; FI: fluorescence intensity; NFI: net fluorescence intensity; HLPP: Human Liver Proteome Project.

## Competing interests

The authors declare that they have no competing interests.

## Authors' contributions

QML conceived of the study and wrote the manuscript. QC carried out the main work of MSIP setup. JW provided large amounts of PPI candidates. Y Zhang and Y Zhou performed the traditional resin-based coimmunoprecipitation assay. CL and WH help to set up MSIP and analyze the data. FCH and DKX supervised the whole work. All authors read and approved the final manuscript.

## Supplementary Material

Additional file 1 **Supporting information**. The information of all the protein pairs analyzed by MSIP. Click here for file
