# Supplementary material for: A miniaturized sandwich immunoassay platform for the detection of protein-protein interactions
Source: BMC Biotechnol. 2010 Oct 28;10:78. doi: 10.1186/1472-6750-10-78 (PMC2978116; doi:10.1186/1472-6750-10-78)
Supplement: Additional file 1 — Supporting information. The information of all the protein pairs analyzed by MSIP. [file 1472-6750-10-78-S1.DOC]

# SUPPORTING INFORMATION

**Table 1. Evaluation of the specificity of PPI determination by MSIP**. 6 pairs of well-characterized interacting proteins and 4 pairs of known non-interacting proteins were assessed by MSIP, as described in *Methods*. Net Fluorescence Intensity was determined for each interaction. Each positive PPI generated a NFI value of ≥243.00; SD, standard deviation.

| **Interaction** | | **Net Fluorescence Intensity** | | | | | | **Reference** |
| --- | --- | --- | --- | --- | --- | --- | --- | --- |
| **FLAG-X** | **Myc-Y** | **slide 1** | | **slide 2** | | **slide 3** | |  |
|  |  | **average** | **SD** | **average** | **SD** | **average** | **SD** |  |
| c-Jun | c-term2 | -82.33 | 10.41 | -52.67 | 9.17 | -49.83 | 14.00 | *EMBO J*. 2005;24(4):766-78. |
| c-Jun | △PH | -84.67 | 10.41 | -47.33 | 4.36 | -22.00 | 4.04 | *EMBO J*. 2005;24(4):766-78. |
| c-Jun | lacZ | -59.67 | 11.93 | -60.67 | 7.55 | -54.67 | 2.65 | *EMBO J*. 2005;24(4):766-78. |
| CKIP-1 | LZPR | 6.33 | 11.59 | -2.33 | 1.00 | 6.33 | 8.54 | *EMBO J*. 2005;24(4):766-78. |
| c-Jun | CKIP-1 | 307.50 | 158.03 | 308.00 | 49.54 | 243.00 | 24.88 | *EMBO J*. 2005;24(4):766-78. |
| MafG | Nrf2 | 1478.33 | 217.95 | 1299.67 | 3.79 | 1548.67 | 179.70 | *J Biol Chem*. 2000; 275(51):40134-41. |
| Keap1 | Nrf2 | 1722.67 | 56.36 | 2409.33 | 140.16 | 2963.33 | 278.75 | *Genes Dev*. 1999; 13(1):76-86. |
| MafK | Nrf2 | 1243.33 | 107.68 | 1240.67 | 111.47 | 821.67 | 102.45 | *J Biol Chem*. 2000; 275(51):40134-41. |
| TRB3 | ATF4 | 2108.67 | 147.93 | 1049.67 | 159.64 | 1059.00 | 69.08 | *EMBO J*. 2005; 24(6):1243-55. |
| NF-κB1 | RelA | 12412.33 | 198.56 | 11105.33 | 234.70 | 11145.67 | 438.95 | *EMBO J*. 1991; 10(7):1817-25. |

**Table 2. Evaluation of the NFI for PPIs using TRB3 as bait**. NFI was determined for each of 8 potential interacting partners with TRB3, by MSIP, as described in *Methods*. NFI, net fluorescence intensity; SD, standard deviation.

| **FLAG-X** | **Myc-Y** | **NFI** | **SD** |
| --- | --- | --- | --- |
| TRB3 | Myo18a | -19.00 | 10.21 |
| TRB3 | GRN | 166.67 | 21.46 |
| TRB3 | ATF5 | 561.00 | 76.54 |
| TRB3 | MCM3AP | 1526.00 | 166.41 |
| TRB3 | FN1 | 3231.00 | 403.51 |
| TRB3 | LTBP4 | 9102.33 | 571.07 |
| TRB3 | CSNK2B | 1894.83 | 43.14 |
| TRB3 | ATF4 | 15171.17 | 1973.16 |

**Table 3. The biological replicates tested by MSIP. The biological replicates of 6 positives and 4 negatives were tested by MSIP on 3 slides, as described in *Methods*. Net Fluorescence Intensity was determined for each interaction. SD, standard deviation; CV, coefficient of variation.**

| Interaction | | **Net Fluorescence Intensity** | | | Average | SD | CV(%) |
| --- | --- | --- | --- | --- | --- | --- | --- |
| **FLAG-X** | **Myc-Y** | **slide 1** | **slide 2** | **slide 3** |
| c-Jun | c-term2 | -82.33 | -52.67 | -49.83 | — | — | — |
| c-Jun | △PH | -84.67 | -47.33 | -22.00 | — | — | — |
| c-Jun | lacZ | -59.67 | -60.67 | -54.67 | — | — | — |
| CKIP-1 | LZPR | 6.33 | -2.33 | 6.33 | — | — | — |
| c-Jun | CKIP-1 | 307.50 | 308.00 | 243.00 | 286.17 | 37.38 | 13.06 |
| MafG | Nrf2 | 1478.33 | 1299.67 | 1548.67 | 1442.22 | 128.37 | 8.90 |
| Keap1 | Nrf2 | 1722.67 | 2409.33 | 2963.33 | 2365.11 | 621.51 | 26.28 |
| MafK | Nrf2 | 1243.33 | 1240.67 | 821.67 | 1101.89 | 242.68 | 22.02 |
| TRB3 | ATF4 | 2108.67 | 1049.67 | 1059.00 | 1405.78 | 608.74 | 43.30 |
| NF-κB1 | RelA | 12412.33 | 11105.33 | 11145.67 | 11554.44 | 743.23 | 6.43 |

**Table 4: Assessment of the reproducibility of PPI determinations by MSIP**. Net fluorescence intensity was determined following incubation of increasing amounts of cellular lysates (5-50 μg). Interactions between both NF-κB + RelA and TRB3 + FN1 were determined by MSIP. MSIP was performed as described in *Methods*.

| **Net Fluorescence Intensity** | | | | | | |
| --- | --- | --- | --- | --- | --- | --- |
| **Cell lysates**  **(μg)**  **Interaction** | **5** | **10** | **20** | **30** | **40** | **50** |
| **NF-κB+RelA** | 2705.00 | 5297.17 | 10014.83 | 15923.67 | 16390.67 | 20357.17 |
| **TRB3+FN1** | 131.83 | 327.50 | 702.00 | 1666.83 | 3130.67 | 3332.33 |

**Table 5. Determination of NFI cutoff value for automated detection of positive PPIs.** 52 pairs of FLAG-bait and myc-prey were randomly selected and analyzed by MSIP, as described in *Methods*. The light shaded boxes indicate negative FI values that were not included in the determination of NFI cutoff value. To generate a more stringent cutoff, the cutoff value was calculated following elimination of data (dark shaded boxes) in which a negative NFI value was observed. The cutoff value of NFI was defined as mean value plus double standard error of the remaining values, and calculated to be 151.2. FI, fluorescence intensity; NFI, net fluorescence intensity

| **No.** | **FLAG-X** | **Myc-Y** | **FI of sample** | **FI of control** | **NFI** |
| --- | --- | --- | --- | --- | --- |
| **1** | P50 | ATF4 | 51.33 | 40.50 | 10.83 |
| **2** | P50 | Jlip | 127.33 | 70.17 | 57.17 |
| **3** | P50 | c-term1 | -33.67 | 45.00 | -78.67 |
| **4** | P50 | FN1 | -98.83 | -25.67 | -73.17 |
| **5** | P50 | MCM3AP | -23.67 | -81.00 | 57.33 |
| **6** | P50 | ATF5 | 190.50 | 44.33 | 146.17 |
| **7** | P50 | C-term2 | -105.17 | 63.00 | -168.17 |
| **8** | P50 | LZPR | -130.67 | -45.33 | -85.33 |
| **9** | P50 | lacZ | 47.67 | -133.33 | 181.00 |
| **10** | P50 | △PH | -31.33 | -74.67 | 43.33 |
| **11** | TRB3 | Jlip | 121.50 | 44.33 | 77.17 |
| **12** | TRB3 | c-term1 | -171.67 | -10.67 | -161.00 |
| **13** | TRB3 | C-term2 | -192.00 | 32.33 | -224.33 |
| **14** | TRB3 | LZPR | -104.33 | -19.33 | -85.00 |
| **15** | TRB3 | △PH | -75.33 | -9.33 | -66.00 |
| **16** | MafG | P65 | -226.67 | -7.83 | -218.83 |
| **17** | MafG | Jlip | 6.00 | 18.67 | -12.67 |
| **18** | MafG | c-term1 | 154.00 | 21.33 | 132.67 |
| **19** | MafG | FN1 | -2.67 | -64.00 | 61.33 |

Table 5 (continued)

| **No.** | **FLAG-X** | **myc-Y** | **FI of sample** | **FI of control** | **NFI** |
| --- | --- | --- | --- | --- | --- |
| **20** | MafG | MCM3AP | -155.67 | -24.00 | -131.67 |
| **21** | MafG | ATF5 | -99.67 | -92.33 | -7.33 |
| **22** | MafG | C-term2 | 16.33 | -43.67 | 60.00 |
| **23** | MafG | LZPR | -32.67 | -19.67 | -13.00 |
| **24** | MafG | lacZ | -77.33 | -26.67 | -50.67 |
| **25** | MafG | △PH | -145.17 | -63.33 | -81.83 |
| **26** | Jun | P65 | -229.00 | -11.67 | -217.33 |
| **27** | Jun | ATF4 | -148.67 | -73.33 | -75.33 |
| **28** | Jun | Jlip | 159.33 | 125.33 | 34.00 |
| **29** | Jun | C-term2 | 13.00 | 5.33 | 7.67 |
| **30** | Jlip | LZPR | 64.67 | 80.33 | -15.67 |
| **31** | Jlip | lacZ | 18.33 | -7.67 | 26.00 |
| **32** | Jun | △PH | 92.00 | 108.33 | -16.33 |
| **33** | Jun | lacZ | 112.67 | 100.00 | 12.67 |
| **34** | TRB3 | Myo18A | 61.00 | 80.00 | -19.00 |
| **35** | TRB3 | lacZ | -140.67 | -108.50 | -32.17 |
| **36** | MafG | ATF4 | -64.33 | -59.33 | -5.00 |
| **37** | SMAD3 | PPP1R12C | 183.00 | 178.00 | 5.00 |
| **38** | SMAD3 | RNF31 | 79.00 | 60.67 | 18.33 |
| **39** | SMAD3 | ZC3H12A | -9.00 | 13.67 | -22.67 |
| **40** | SMAD3 | SETD2 | 39.33 | 51.33 | -12.00 |
| **41** | SMAD4 | NLK | 119.67 | 30.67 | 89.00 |
| **42** | NR4A1 | GK | -5.17 | 137.00 | -142.17 |
| **43** | GK | NR4A1 | 29.00 | 61.67 | -32.67 |

Table 5 (continued)

| **No.** | **FLAG-X** | **myc-Y** | **FI of sample** | **FI of control** | **NFI** |
| --- | --- | --- | --- | --- | --- |
| **44** | SETD7 | STEAP3 | 102.33 | 61.00 | 41.33 |
| **45** | PML(2-143) | CASP8AP2 | 194.33 | 130.33 | 64.00 |
| **46** | PLK1 | OFD1 | 44.00 | 38.00 | 6.00 |
| **47** | TP73L(501-678) | CCNC | 87.33 | 63.67 | 23.67 |
| **48** | RCHY1 | CHD8 | 146.67 | 119.33 | 27.33 |
| **49** | CTBP2 | XRCC6 | 280.67 | 184.33 | 96.33 |
| **50** | PIN1 | MOCS1 | 64.67 | 60.33 | 4.33 |
| **51** | PIN1 | SUPT5H | 104.33 | 77.00 | 27.33 |
| **52** | TXN2 | FN1 | 209.00 | 133.67 | 75.33 |

**Table 6. Comparison of PPI identification by MSIP versus resin-based coIP.** 18 pairs of interaction candidates identified by yeast two-hybrid screening were analyzed for PPI by both MSIP and traditional resin-based coIP. All the interactions had LHU (L, *lacZ*; H, *HIS3*; U, *URA3*) Phenotype in YTH assay. NFI values above the cutoff value of 151.2, determined in Table S4, are considered positive. The interactions of PIRH2 with SERPINA1 and PIRH2 with PMM1 were identified as the positive by MSIP not confirmed by traditional coIP. The small numbers refer to the amino acid range of the corresponding protein construct. NFI, net fluorescence intensity; SD, standard deviation

| **FLAG-X** | **Myc-Y** | **NFI** | **SD** | **Resin-based coIP** |
| --- | --- | --- | --- | --- |
| ARF | TDRD7 | 817.83 | 166.61 | Y |
| ARF | HNRPU | 2132.33 | 114.61 | Y |
| ARF | DEAF1 | 1049.33 | 97.57 | Y |
| ARF | CRELD2 | 34123.67 | 855.11 | Y |
| PIRH2 | HRG | 2751.33 | 669.23 | Y |
| PIRH2 | PMM1 | 13803.00 | 548.28 | N |
| TP73L^501-678^ | CCNC | 13.67 | 6.43 | N |
| TP73L^501-678^ | GCGR | -23.00 | 10.44 | N |
| PML^2-143^ | PSMA3 | 3255.83 | 7.51 | Y |
| PML^2-143^ | CASP8AP2 | 91.67 | 25.15 | N |
| PIRH2 | SERPINA1 | 368.67 | 293.78 | N |
| PIN1 | MAP3K11 | 357.67 | 36.76 | Y |
| CTBP2 | BCL3 | 7042.00 | 173.82 | Y |
| CTBP2 | SOX13 | 574.17 | 128.54 | Y |
| CTBP2 | XRCC6 | 4.33 | 7.51 | N |
| CTBP2 | PPP1R15A | 872.67 | 86.69 | Y |
| CTBP2 | PROX1 | 1860.50 | 178.73 | Y |
| GSK3B | ZFPM1 | 290.17 | 52.60 | Y |

**Table 7. Comparison of MSIP with resin-based coIP**

|  | MSIP | Resin-based coIP |
| --- | --- | --- |
| Number of cells | ~5×10^4^ (in 96-well plate) | ≥3×10^6^ (in 60cm^2^ flask) |
| Time cost | Within 5 hours | ≥1day |
| Procedures | Simple and easy | Complex and tedious |
| Cost | Low (~$1.5/each PPI) | High (>$15.0/each PPI) |
| Large scale analysis of PPIs | Easy | Difficult |

**Table 8. Application of MSIP to verify 48 pairs of PPI candidates from YTH system**. 48 additional pairs of interaction candidates identified by yeast two-hybrid (YTH) screening were analyzed for PPI by both MSIP. NFI values above the cutoff value of 151.2, determined in Table S4, are considered positive. 27 of the 48 pairs identified by YTH screening were determined to be negative for PPI by MSIP. NFI, net fluorescence intensity; SD, standard deviation.

| **No.** | **FLAG-X** | **myc-Y** | **NFI** | **SD** | **Interaction** |
| --- | --- | --- | --- | --- | --- |
| **1** | KPNA2 | JUN | 814.83 | 14.29 | Y |
| **2** | KPNA2 | TRAF1 | 135.00 | 153.58 | N |
| **3** | JAB1 | PRDX2 | 842.50 | 200.10 | Y |
| **4** | JAB1 | p105 | 511.00 | 41.02 | Y |
| **5** | MAP3K8 | MYD88 | 241.17 | 12.75 | Y |
| **6** | MAP2K5 | ESRRA | 717.00 | 245.64 | Y |
| **7** | SMAD3 | SQSTM1 | 1287.17 | 322.01 | Y |
| **8** | SMAD3 | PPP1R12C | -23.00 | 26.89 | N |
| **9** | SMAD3 | RNF31 | 28.00 | 10.54 | N |
| **10** | SMAD3 | ZC3H12A | -22.33 | 16.00 | N |
| **11** | SMAD3 | SETD2 | -1.00 | 11.37 | N |
| **12** | SMAD3 | CHRD | 811.00 | 114.42 | Y |
| **13** | SMAD3 | PCK2 | 452.00 | 120.33 | Y |
| **14** | SMAD3 | TSC22D4 | 1705.33 | 131.33 | Y |
| **15** | SMAD4 | NLK | 85.67 | 15.37 | N |
| **16** | SMAD4 | UBC9 | 451.50 | 144.61 | Y |
| **17** | NR4A1 | GK | -125.50 | 82.05 | N |
| **18** | GK | NR4A1 | -37.33 | 17.78 | N |
| **19** | SQLE | CREB3 | 289.50 | 17.47 | Y |
| **20** | GADD45A | SH3GLB1 | -109.40 | 4.00 | N |
| **21** | GADD45A | NUCB2 | -104.17 | 28.91 | N |

Table 8 (continued)

| **No.** | **FLAG-X** | **myc-Y** | **NFI** | **SD** | **Interaction** |
| --- | --- | --- | --- | --- | --- |
| **22** | PLK1 | PHC2 | 6135.00 | 494.70 | Y |
| **23** | PLK1 | OFD1 | -3.33 | 4.36 | N |
| **24** | PLK1 | TANK | 9574.67 | 605.77 | Y |
| **25** | SIPA1 | CDKN1A | 456.67 | 35.02 | Y |
| **26** | TXNDC11 | CDKN1A | 187.67 | 7.55 | Y |
| **27** | CCNH | GOLGA2 | 166.33 | 53.58 | Y |
| **28** | RCHY1 | CHD8 | 51.00 | 32.72 | N |
| **29** | RCHY1 | ZNF160 | -10.67 | 16.92 | N |
| **30** | PIN1 | TNIP1 | -43.67 | 8.62 | N |
| **31** | PIN1 | MOCS1 | -8.67 | 3.51 | N |
| **32** | PIN1 | SUPT5H | 26.00 | 7.57 | N |
| **33** | KEAP1 | NFE2L1 | 228.00 | 18.15 | Y |
| **34** | KEAP1 | FGA | 1891.83 | 2098.47 | Y |
| **35** | KEAP1 | RELA | 133.00 | 19.14 | N |
| **36** | MafG | NFE2L2 | 386.33 | 25.72 | Y |
| **37** | MafG | CYP2E1 | -3.33 | 12.06 | N |
| **38** | TXN2 | GPRASP2 | 130.67 | 41.80 | N |
| **39** | TXN2 | FN1 | 79.00 | 14.00 | N |
| **40** | TXN2 | ALDOB | 73.33 | 41.68 | N |
| **41** | TXN | RDH11 | 6.00 | 6.35 | N |
| **42** | TXN1 | GRAMD1C | -15.67 | 8.96 | N |
| **43** | ABCC2 | P105/NFKB1 | 148.00 | 55.67 | N |
| **44** | BIK | SOCS3 | -54.33 | 13.00 | N |

Table 8 (continued)

| **No.** | **FLAG-X** | **myc-Y** | **NFI** | **SD** | **Interaction** |
| --- | --- | --- | --- | --- | --- |
| **45** | DAXX | FT1-1 | 359.67 | 111.40 | Y |
| **46** | GADD45A | SH3GLB1 | 71.00 | 12.58 | N |
| **47** | GRB2 | ERRFI1 | 14716.17 | 2484.22 | Y |
| **48** | SETD7 | STEAP3 | 75.33 | 13.71 | N |

**REFERENCES**

1. Zhang L, Xing G, Tie Y, Tang Y, *et al*. (2005) Role for the pleckstrin homology domain-containing protein CKIP-1 in AP-1 regulation and apoptosis. *EMBO J.* 24(4):766-78.
2. Dhakshinamoorthy S, Jaiswal AK. (2000) Small maf (MafG and MafK) proteins negatively regulate antioxidant response element-mediated expression and antioxidant induction of the NAD(P)H:Quinone oxidoreductase1 gene. *J Biol Chem.* 275(51):40134-41.
3. Itoh K, Wakabayashi N, Katoh Y, Ishii T, *et al*. (1999) Keap1 represses nuclear activation of antioxidant responsive elements by Nrf2 through binding to the amino-terminal Neh2 domain. *Genes Dev.* 13(1):76-86.
4. Ohoka N, Yoshii S, Hattori T, Onozaki K, Hayashi H.( 2005) TRB3, a novel ER stress-inducible gene, is induced via ATF4-CHOP pathway and is involved in cell death. *EMBO J*. 24(6):1243-55.
5. Urban MB, Schreck R, Baeuerle PA. (1991) NF-kappa B contacts DNA by a heterodimer of the p50 and p65 subunit. *EMBO J.* 10(7):1817-25.
